# Prince of Wales's Hospital Fund

**Published:** 1897-12-25

**Authors:** 


### PRINCE OF WALES'S HOSPITAL FUND.

### GENERAL MEETING OF THE COUNCIL.

A general meeting of the Council of the Prince of Wales's Hospital Fund for London was held on Monday, 20th inst., at Marlborough House. The Prince of Wales, President of the Fund, was in the chair, and there were also present the Duke of Norfolk, the Bishop of London, the Bishop of Rochester, the Rev. Dr. Guinness Rogers, the Chief Rabbi, Lord Rowton, Lord Rothschild, Lord Iveagh, the Lord Mayor, Dr. Collins, Mr. Hugh Colin Smith, Lord Lister, Sir Samuel Wilks, Bart., the Right Hon. C. Stuart Wortley, Q.C., M.P., Sir Horace Farquhar, Bart., M.P., Sir Henry Burdett, K.C.B., Mr. John Aird, M.P., Mr. Sydney Buxton, M.P., Mr. E. A. Hambro, Mr. Albert G. Sandeman, Mr. Julius Wernher, Sir Savile Crossley, Bart., and Mr. Ian Malcolm, M.P. (hon. secretaries), and Mr. J. G. Craggs (hon. assistant secretary). Letters of regret were read from the Duke of Westminster, the Earl of Strafford, Cardinal Vaughan, the Rev. Dr. Stevenson, Lord Reay, Sir William MacCormac, and Sir Arthur Arnold.

#### Financial.

Lord Rothschild, as treasurer of the Fund, presented his financial statement. He said that there was at the present moment at the Bank of England and other banks a sum of £187,000, of which £20,500 had been received as annual subscriptions and £1,500 as interest from moneys invested; so that the income of the Fund so far had amounted to about £22,000. In addition, the Fund would receive towards the end of the year £38,000 to £40,000 from the sale of the hospital stamps. Altogether, therefore, His Royal Highness would have at his disposal, to give to the hospitals, not for this year, but he hoped in perpetuity, a sum of £22,000, and in addition to this he would have from £38,000 to £40,000 to give to all the hospitals as a special donation this year in commemoration of her Gracious Majesty's Jubilee. In addition to these amounts he (the speaker) hoped that in a very few days they would hear that the great City companies had followed the example of the Fishmongers' Company, who had voted a grant of £1,000 a year for five years to the Fund.

Mr. Hugh Colin Smith presented the report of the executive committee, which, on the motion of Mr. John Aird, M.P., seconded by the Lord Mayor, was adopted.

#### The Central Hospital Board.

Mr. John Aird, M.P., moved:

"That this Council hereby approves and adopts the resolution passed by the executive committee on November 9th, 1897, that, in view of the controversial character of the proposition of the Charity Organisation

Society to establish a central hospital board for London, it is inexpedient that the Prince of Wales's Hospital Fund for London be associated with the movement."

The Duke of NORFOLK seconded, and the resolution was agreed to.

QUEEN VICTORIA'S JUBILEE NURSING INSTITUTION.

Sir SAVILE CROSSLEY, Bart., read a letter from the Duke of Westminster, dated December 13th, 1897, in which his Grace asked for the consideration by the committee of the claims of the Queen's Jubilee Institution for Nursing the Sick Poor in their own homes for an annual grant from the Fund.

The Bishop of LONDON moved, and the CHIEF RABBI seconded, the following resolution, which was carried:

"That this Council, while thoroughly approving of the work of the Queen Victoria's Jubilee Institute for Nurses, regrets that no part of this Fund can be rightly allocated to the Duke of Westminster's scheme."

THE DISTRIBUTION OF THE FUND.

Lord LISTER presented the report of the Executive Committee in reference to the distribution of the Fund this year. The report recommended that, chiefly with the object of meeting the special wants of the leading hospitals, the sum of £22,050 out of the income of the Fund be distributed amongst some of the larger hospitals, and a sum of £38,000, being approximately the amount derived from the hospital stamps, be distributed amongst certain hospitals in the manner adopted by the Hospital Sunday Fund, but with such modifications as the Special Committee on this subject may, with the approval of the Executive Committee, think it desirable to make. Speaking of the future of the Fund, Lord Lister said that there was reason to hope that many persons who had given donations in the present year would continue these donations in future years, provided it was known that the Prince of Wales's Fund was doing really good work. Further, it was felt that the humbler classes—persons in the middle and lower classes of life, who derived benefit from hospitals, and who were able to give small sums, which, taken on the aggregate of the London population, would amount to a large sum of money—had certainly not been sufficiently attacked, so to speak, and that the Fund might expect very considerable sums in future years from this source. The Fund only professed in this present year to have touched on the question of distribution in a tentative manner, and as it had been quite impossible to go into the circumstances, the merits, and the needs of the smaller hospitals this year, this had been deferred to future years. It had not been left out of view; it had only been physically impossible to do it this year. The committee had, however, been able to consider the needs of the principal hospitals of London, and to those it was proposed to devote the income of the present year. The committee was exceedingly glad to find that if in future years the same could be done as had been done this year, the Prince of Wales's great object of relieving the metropolitan hospitals of their financial embarrassments would be thoroughly and effectively carried out. He moved that the recommendations of the Executive Committee be approved, and that the proposed scheme for distribution be adopted.

The PRINCE OF WALES said: Most of you are aware that my great object has been to be very careful of the moneys entrusted to our charge. I mean by this, that I should deplore if, for the sake of making a great effect before the public by giving a large sum away, we should encroach upon our capital. I am ready to spend every sixpence of our income, but I confess it was with great reluctance that I gave way on the point of spending any of our capital. At the same time, the question of the Hospital stamps, which will realise about £40,000, is a special thing, and I think we may put this amount aside from the sums we have received from other institutions and from other people. I agree with Lord Lister that, in order to have done something towards carrying out the object we had in view, we should this year give a sufficient sum of money to open wards and beds now closed. That is the great point. We must, I think, leave ourselves in the hands of the committee, who have taken such great trouble, in this matter of distribution. If the General Council agrees to the committee's recommendations, I can hardly do otherwise than coincide with their views. My own view on distribution is, that searching inquiries should be made into all matters concerning each hospital, so that all the amounts spent may be wisely administered. Unfortunately, as you must be well aware, in many cases wings and wards are now built when the hospitals can only hope that future charity, by means of dinners and bazaars, may give them the pecuniary assistance necessary to defray expenses; and if they do not receive as much money as the managers hope from these bazaars and dinners, they are ultimately obliged to close the very wards which they recently opened. It is this extravagance in the maintenance of hospitals which this Fund should endeavour to discourage.

Lord IVEAGH seconded the adoption of the report and the approval of the scheme for distribution; and the motion was carried.

LOCAL COMMITTEES.

On the motion of Sir SAVILE CROSSLEY, Bart., seconded by the BISHOP OF LONDON, it was resolved that the Council viewed with great satisfaction the efforts made in Marylebone, Paddington, and elsewhere to establish local committees for the collection of funds for the Prince of Wales's Hospital Fund for London, and trusted that the system would be extended in future years to other parts of the metropolis.

